# Supplementary material for: Investigation of Root Morphological Traits Using 2D-Imaging among Diverse Soybeans (Glycine max L.)
Source: Plants (Basel). 2021 Nov 21;10(11):2535. doi: 10.3390/plants10112535 (PMC8622990; doi:10.3390/plants10112535)
Supplement: Supplementary file 1 [file plants-10-02535-s001.zip › plants-1387415-SI.pdf]

plants

Supplementary table and figure

Table S1. List of soybean genotypes

Cul. No.	IT No.	Region	Cul. No.	IT No.	Region	Cul. No.	IT No.	Region	Cul. No.	IT No.	Region	Cul. No.	IT No.	Region
1	21578	KOR	76	24364	USA	151	163458	CAN	226	165252	CHN	301	196403	CHN
2	21595	USA	77	24367	USA	152	163465	USA	227	165254	CHN	302	196405	CHN
3	21596	USA	78	24368	USA	153	163466	USA	228	165255	CHN	303	196407	CHN
4	21597	USA	79	24376	USA	154	164731	JPN	229	165258	CHN	304	199083	RUS
5	21605	JPN	80	24681	USA	155	165014	USA	230	165263	CHN	305	199084	RUS
6	21617	USA	81	100868	JPN	156	165019	USA	231	165265	CHN	306	199091	RUS
7	21636	USA	82	100869	JPN	157	165020	USA	232	165266	CHN	307	199092	RUS
8	21639	CAN	83	100871	JPN	158	165021	USA	233	165268	CHN	308	199094	RUS
9	21641	USA	84	120656	TWN	159	165022	USA	234	165269	CHN	309	199095	MDA
10	21684	USA	85	134349	KOR	160	165023	USA	235	165270	CHN	310	199097	RUS
11	21685	USA	86	134351	KOR	161	165035	USA	236	165271	CHN	311	199116	PRK
12	21708	KOR	87	135742	JPN	162	165038	USA	237	165277	CHN	312	199117	PRK
13	21710	USA	88	135747	JPN	163	165048	USA	238	165278	CHN	313	199124	PRK
14	21725	JPN	89	135748	JPN	164	165053	USA	239	165280	CHN	314	199125	PRK
15	21761	USA	90	135752	JPN	165	165055	USA	240	165282	CHN	315	199126	PRK
16	21764	USA	91	142750	USA	166	165059	USA	241	165284	CHN	316	199127	PRK
17	21812	USA	92	142757	USA	167	165060	USA	242	165285	CHN	317	199128	PRK
18	22036	USA	93	142759	USA	168	165062	USA	243	165286	CHN	318	199130	KOR
19	22044	USA	94	142794	USA	169	165063	USA	244	165290	CHN	319	199134	PRK
20	22046	USA	95	142797	JPN	170	165066	USA	245	165293	CHN	320	199135	PRK
21	22049	JPN	96	142798	JPN	171	165070	USA	246	165294	CHN	321	199139	PRK
22	22069	USA	97	142804	USA	172	165071	USA	247	165295	CHN	322	199144	RUS
23	22079	USA	98	142807	KOR	173	165076	USA	248	165296	CHN	323	199145	RUS
24	22089	USA	99	142809	KOR	174	165081	USA	249	165297	CHN	324	199149	UKR
25	22109	USA	100	142810	KOR	175	165084	USA	250	165306	CHN	325	199151	RUS
26	22111	USA	101	142811	KOR	176	165087	USA	251	165308	CHN	326	199155	RUS
27	22116	CAN	102	142825	USA	177	165095	CAN	252	165310	CHN	327	199159	MDA
28	22123	USA	103	142828	USA	178	165097	CAN	253	165321	CHN	328	202113	USA
29	22148	USA	104	142873	USA	179	165103	USA	254	165325	CHN	329	203561	KOR
30	22167	USA	105	142888	CHN	180	165104	USA	255	165333	CHN	330	203565	KOR
31	22168	CAN	106	142900	USA	181	165105	USA	256	165337	CHN	331	203568	KOR
32	22178	USA	107	142907	USA	182	165111	CAN	257	165379	UZB	332	203593	JPN
33	22193	USA	108	146112	TWN	183	165113	CAN	258	165405	CHN	333	208248	USA
34	22194	USA	109	146115	TWN	184	165115	USA	259	165410	CHN	334	208253	JPN
35	22218	USA	110	153344	KOR	185	165125	USA	260	165411	CHN	335	208266	ARG
36	22224	USA	111	153934	KOR	186	165126	USA	261	165412	CHN	336	208267	USA
37	22293	USA	112	154762	JPN	187	165127	USA	262	165414	CHN	337	208276	CHN
38	22294	CAN	113	154764	KOR	188	165128	USA	263	165423	CHN	338	208289	CHN
39	22297	USA	114	154767	KOR	189	165129	USA	264	165425	CHN	339	208290	CHN
40	22314	USA	115	156011	JPN	190	165131	USA	265	165426	CHN	340	208294	CHN
41	22341	USA	116	156099	JPN	191	165132	USA	266	165428	CHN	341	208307	IND
42	22691	JPN	117	156106	CHN	192	165134	USA	267	165431	CHN	342	208308	CAN
43	22711	USA	118	156277	CHN	193	165136	USA	268	165432	CHN	343	208311	CHN
44	22721	USA	119	156289	JPN	194	165141	USA	269	165434	CHN	344	208312	CHN
45	22754	JPN	120	157351	JPN	195	165142	USA	270	165437	CHN	345	208314	USA
46	22763	JPN	121	157853	IND	196	165143	USA	271	165438	CHN	346	208316	ISR
47	22773	USA	122	157951	KOR	197	165148	USA	272	165441	CHN	347	208327	RUS
48	22782	JPN	123	158021	CHN	198	165151	USA	273	165835	CHN	348	208329	RUS
49	22784	JPN	124	158078	BIH	199	165158	USA	274	165839	CHN	349	208330	RUS
50	23074	USA	125	158080	CHN	200	165160	USA	275	165841	CHN	350	208334	RUS
51	23093	USA	126	158092	USA	201	165161	USA	276	170844	USA	351	208341	RUS
52	23100	JPN	127	160024	USA	202	165162	USA	277	170861	USA	352	208343	RUS
53	23102	USA	128	160657	CHN	203	165163	USA	278	170871	USA	353	208349	RUS
54	23112	JPN	129	160660	CHN	204	165165	CHN	279	170894	USA	354	209178	KOR
55	23114	USA	130	160668	CHN	205	165168	CHN	280	170900	USA	355	211805	CHN

56	23121	JPN	131	160776	USA	206	165170	CHN	281	171092	CHN	356	211807	CHN
57	23305	JPN	132	160790	USA	207	165176	CHN	282	171163	KGZ	357	211824	CHN
58	23312	JPN	133	160872	USA	208	165184	CHN	283	171177	CHN	358	211825	CHN
59	23499	USA	134	160941	USA	209	165192	CHN	284	173044	BEL	359	211827	CHN
60	23532	USA	135	160949	USA	210	165208	CHN	285	179809	UKR	360	211830	CHN
61	23569	USA	136	160951	CHN	211	165210	CHN	286	180383	USA	361	212816	USA
62	23593	USA	137	160989	USA	212	165211	CHN	287	180393	JPN	362	212818	USA
63	23756	USA	138	161000	USA	213	165215	CHN	288	180395	JPN	363	212822	CHN
64	23763	USA	139	161396	USA	214	165217	CHN	289	180396	JPN	364	212824	CHN
65	23781	USA	140	162707	USA	215	165219	CHN	290	180400	JPN	365	212825	CHN
66	23783	USA	141	162973	USA	216	165221	CHN	291	180415	USA	366	212826	CHN
67	24181	USA	142	162992	CHN	217	165222	CHN	292	180429	CHN	367	212827	CHN
68	24188	USA	143	163438	USA	218	165224	CHN	293	181031	JPN	368	212843	CHN
69	24192	USA	144	163447	USA	219	165225	CHN	294	181034	JPN	369	212849	CHN
70	24258	USA	145	163448	USA	220	165226	CHN	295	181348	KOR	370	212850	CHN
71	24282	GEO	146	163450	USA	221	165231	CHN	296	185227	TWN	371	212860	KOR
72	24283	GEO	147	163451	USA	222	165233	CHN	297	185275	CHN	372	212861	KOR
73	24284	GEO	148	163452	USA	223	165237	CHN	298	186742	JPN			
74	24350	USA	149	163453	USA	224	165240	CHN	299	189209	KOR			
75	24361	USA	150	163457	USA	225	165249	CHN	300	189210	KOR			

In the table, Cul. and No. meant cultivars and numbers, respectively.

Figure S1. Root images of contrasting soybean genotypes.

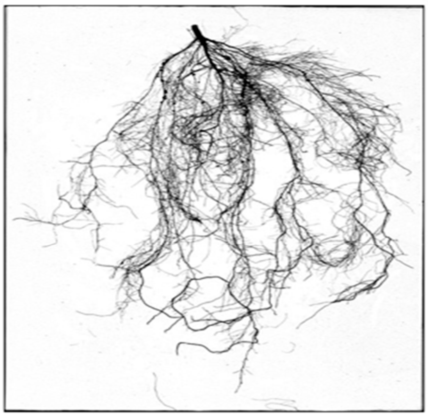

IT 165308

Traits	Values
TL (cm)	777.956
SA (cm^2)	130.721
AD (mm)	0.533
LAL (cm)	0.329
LAD (mm)	0.582
LABL (°)	55.137

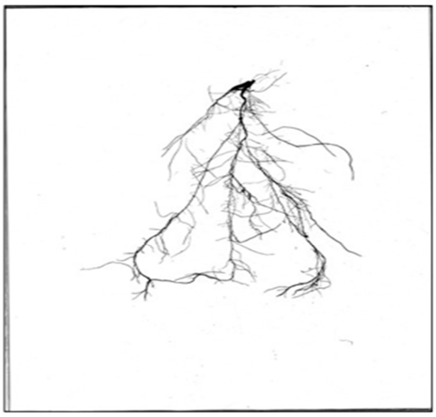

IT 165019

Traits	Values
TL (cm)	113.039
SA (cm^2)	23.675
AD (mm)	0.785
LAL (cm)	0.270
LAD (mm)	0.873
LABL (°)	53.033

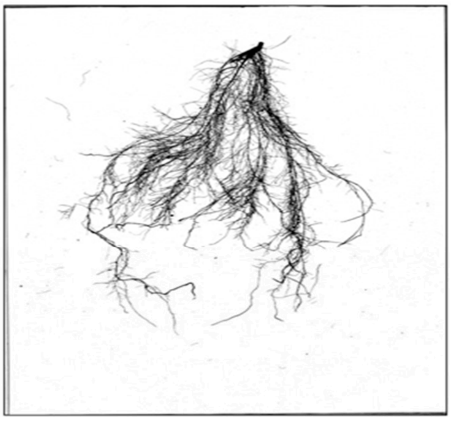

IT 165282

Traits	Values
TL (cm)	534.736
SA (cm^2)	82.228
AD (mm)	0.925
LAL (cm)	0.590
LAD (mm)	1.070
LABL (°)	52.460

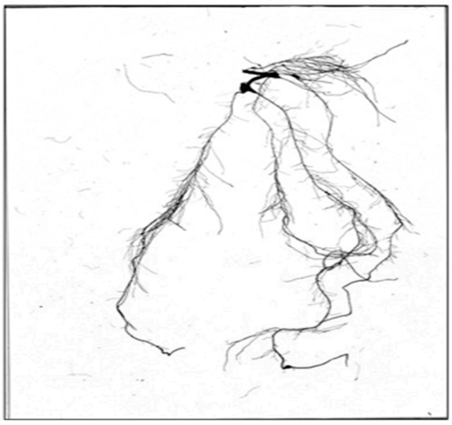

IT 165208

Traits	Values
TL (cm)	127.31
SA (cm^2)	29.607
AD (mm)	0.518
LAL (cm)	0.470
LAD (mm)	0.577
LABL (°)	52.965

Note: Total length (TL), average diameter (AD), surface area (SA), link average length (LAL), link average diameter (LAD), and link average branching angle and (LABA).
